# Books Reviewed

**Published:** 1868-03

**Authors:** 


### Editorial Department.

### Books Reviewed.

Diseases of the Lungs and Air-Passages. By Henry Wm. Fuller, M. D. From the second and revised London edition. Philadelphia: Henry C. Lea, 1867.

Commencing with the principles of physical diagnosis, and their application to the investigation of diseases of the lungs, the author proceeds to a complete discussion of all subjects pertaining to diseases of the respiratory organs and their proper treatment. He gives the topography of the walls, and describes and illustrates with wood cuts the contents of the various regions of the chest; shows the importance and value of inspection and physical examination of the chest, and describes the methods of making these examinations; points out most clesrly what is to be observed and the import and significance of symptoms elicited from the alterations which the various organs undergo in disease.

In part second we have the Pathology, Diagnosis, Symptoms and Treatment of Diseases of the Lungs. Pleurisy, Pneumonia, Bronchitis and Pulmonary Consumption are the diseases which have received principal attention. All the numerous questions concerning tubercular disease, not yet settled, are discussed, and the opinions of the author and the facts sustaining his opinions are introduced. Hereditary transmission of the disease, age at which consumption occurs, influence of cold and wet and the atmospheric changes in producing or predisposing to it, furnish favorite topics, upon which the author entertains opinions not wholly in harmony with the generally received doctrines of authors or the public. Upon one single point we quote our author, because we can so heartily endorse most of his views, and have often presented and urged the same against strong opposition. "Fistula in ano is another symptom which must not be lightly dealt with.

As long as the discharge is insignificant in amount, it is advisable to confine our efforts to treatment of the constitutional malady, and not to disturb the fistula. But I do not hold with those who maintain that a fistula in ano occurring in the course of phthisis ought never to be interfered with. In some instances the discharge is profuse, and constitutes an important source of waste; the patient is so distressed and alarmed at its continuance, that no treatment can be of avail until his nervous apprehensions are overcome. He can neither eat nor sleep for thinking of it, and his whole system is depressed in consequence. In such cases I have known the greatest benefit result from an operation, combined with an issue in the arm, the use of proper diet and administration of cod-liver oil, quinine, iron and other appropriate remedies. Not only has the fistula healed, but the general health has improved, the patient gained flesh, and the physical signs of pulmonary disease have greatly lessened." If the statement of "combined with an issue in the arm" had been omitted, the sentiment would have been worthy a recent author. This part of it we believe the effect of routine in sentiment and practice, and such issue useless and injurious—injurious in proportion to its size and the irritation and ulceration it occasions.

Our new books are better than our old ones, our knowledge is greater and more definite, and our practice of medicine and views of disease are constantly growing more correct and satisfactory, but the mistakes and errors of the past, however clearly pointed out and well defined, are rejected and abandoned only by degrees. The author of this book has done his part faithfully, and furnished the medical public a clear, philosophical, correct and valuable work upon diseases of the lungs and air-passages. It has many attractions for the medical student,in its first part especially, while the practical portion of the work as embodied in its second division, renders it a valuable guide in the treatment of all diseases of the lungs.

---

A Practical Treatise on the Diseases of Children. By D. Francis Condie, M. D., Fellow of the College of Physicians, Member of the American Medical Association, etc., etc. Sixth edition, revised and enlarged. Philadelphia: Henry C. Lea, 1868.

Dr. Condie's Treatise on Diseases of Children is so favorably known and so highly appreciated by the profession, that any extended notice of it is altogether unnecessary, while declarations of approval could be only a repetition of commendations already many times expressed. The present edition has again been thoroughly revised and every important advance in the knowledge of infantile disease has been incorporated in the respective sections, so that as the work now appears it will continue to be an accurate and faithful guide in the treatment of children, and be regarded as one of the most complete works in this department of medicine. Much attention in the commencement of the work has been devoted to a consideration of the hygienic management of children, which embraces a discussion of the influence of light, temperament, cleanliness, bathing, clothing, food, sleep, exercise and moral treatment. It is remarkable that in the face of the acknowledged influence of hygienic regulations as a preventive of disease, and the neglect of which is the cause of nearly all the ails of childhood, physicians should fail to instruct and impress those in charge of infants with the importance of a strict compliance with sanitary laws, and not as is but too frequently the case, through carelessness and sometimes ignorance, allow mothers and nurses to subject these tender beings to the most pernicious influences.

In treating of congenital malformations and accidents, the author entertains a not commonly received view of the pathological cause giving rise to the non-development of the lateral processes of the vertebræ in spina bifida. The disease he considers as a true congenital dropsy either of the spine or of the spine and brain; "*the deficiency in the vertebræ,*' as well as the exterual tumor, being the result of the acuumulation and *pressure* of the fluid within the spinal or cranial cavity, and that when the tumor in the spine is not formed, death usually occurs within a month, with symptoms of hydrocephalus.

---

Pennsylvania Hospital Reports, Vol. 1, 1868. Philadelphia: Lindsay & Blakiston.

The inauguration of publishing annual volumes of Hospital Reports, thus placing the acquired experience of hospital practice upon a permanent record, and making it possible for the profession at large to avail itself of the same, cannot fail to meet with universal approbation and support. Works of this character have been issued by the principal hospitals of England and upon the continent with great success. This is the first effort of the kind in this country, so far as we are informed, but we are assured that reports from some others may soon be axpected.

The present volume embraces twenty-three articles, prepared by the hospital staff, arranged by Drs. J. M. Da Costa and William Hunt.

The introductory paper prepared by Dr. Charles D. Meigs, is a brief sketch of the history of the Pennsylvania hospital and reminiscences of the physicians and surgeons who served in the same. Dr. H. Agnew follows with a careful and well-considered paper on the history and treatment of laceration of the female perineum. The other members of the staff have equally well discharged their tasks, presenting in an exact statement the experience of the past years in their respective departments. The action of Narcine has been made the subject of a monograph by Dr. J. M. Da Costa, from whose paper we make the following extract:

"On the skin it produces but little effect, far less perspiration than morphia or the other ingredients of opium.

It does not, as a rule, give rise to headache, or to nausea and vomiting and loss of appetite; but it is an exaggeration to say that these effects do not occur. Moreover they seem to happen more constantly or markedly in women than in men. It does not constipate, may even relax the bowels.

It is not an excitant; yet the face is not uncommonly flushed after its use in decided doses. Scarcely any action on the pupils is observable.

No marked influence on the temperature, respiration and pulse is perceptible subsequent to its employment. So far as noticed it somewhat lowered the temperature, and slightly lessened the pulse; the latter, however, not constantly.

No such decided effect as has been ascribed to it on the urinary functions, was met with. In so far as it was seen to have any action, it seemed to diminish the tendency to frequent urination, rather than to suppress the amount of secretion.

And with reference to its soporific anodyne properties, it appeared in doses in which morphia is prescribed, totally destitute of either; and in larger doses uncertain and often palpably inert. It does not allay irritation."

What will the French physicians say to this, with whom *Narcine* is fashionable? Dr. Eulenberry prefers it to any other narcotic, and gives it in neuralgia, iritis, cystitis, orchitis, and all painful diseases, stating that it produces sleep, and is preferable to morphia, acting pleasantly when morphia fails.

